# Low-dose *Drosera rotundifolia* induces gene expression changes in 16HBE human bronchial epithelial cells

**DOI:** 10.1038/s41598-021-81843-y

**Published:** 2021-01-27

**Authors:** Fabio Arruda-Silva, Paolo Bellavite, Marta Marzotto

**Affiliations:** Department of Medicine, University of Verona, strada Le Grazie 8, 37134 Verona, Italy

**Keywords:** Transcriptomics, RNA sequencing, Reverse transcription polymerase chain reaction, Therapeutics

## Abstract

*Drosera rotundifolia* has been traditionally used for the treatment of respiratory diseases in phytotherapy and homeopathy. The mechanisms of action recognized so far are linked to the known effects of specific components, such as flavonoids, but are not completely understood. In this study, the biological functions of *D. rotundifolia* were explored in vitro following the treatment of bronchial epithelial cells, which are the potential targets of the pharmacological effects of the herbal medicine. To do so, the whole plant ethanolic extract was 1000-fold diluted in water (*D. rotundifolia* 3×) and added to a 16HBE human cell line culture for 3 h or 6 h. The effects on gene expression of the treatments and corresponding controls were then investigated by RNA sequencing. The differentially expressed genes were validated through RT-qPCR, and the enriched biological functions involved in the effects of treatment were investigated. *D. rotundifolia* 3× did not impair cell viability and was shown to be a stimulant of cell functions by regulating the expression of dozens of genes after 3 h, and the effects were amplified after 6 h of treatment. The main differentially expressed genes encoded ligands of epithelial growth factor receptor, proteins involved in xenobiotic detoxification and cytokines, suggesting that *D. rotundifolia* 3× could stimulate self-repair systems, which are impaired in airway diseases. Furthermore, *D. rotundifolia* 3× acts on a complex and multifaceted set of genes and may potentially affect different layers of the bronchial mucosa.

## Introduction

Pathologies of the airways are among the most common diseases. Cough is a common complaint in patients of all ages, with air pollutants contributing to airway challenge. In children, cough is the second most common symptom of respiratory disease after runny nose, with a 46–56% prevalence depending on the age of the child. The use of pharmacological treatment without side effects could be useful not only to reduce cough symptoms but also to prevent the toxic effects of pollution. Low doses of plant-derived drugs are largely used for cold and cough symptoms in adults and children^1–7^. Often, these are used with the hope of resolving ailments not successfully cured by conventional drugs as a complementary treatment to reduce the consumption of anti-inflammatory drugs or steroids that may have adverse effects and to relieve certain symptoms and improve the quality of life. However, there is presently no definitive explanation for the possible biological activity of such preparations at the cellular level.

European sundew species (*Drosera rotundifolia* L., *Drosera intermedia* Hayne, and *Drosera anglica* Huds) have been used as traditional medicines in the therapy of respiratory tract infections. *Drosera* Herba, which is comprised chiefly of *Drosera rotundifolia*, has been commonly used for its spasmolytic properties in the treatment of convulsive or whooping cough since the seventeenth century. The general action on the respiratory system of *D. rotundifolia* was described in the homeopathic Materia Medica^8^ and includes profuse expectoration and a spasmodic, paroxysmal, dry, and irritative cough, similar to whooping cough, and the plant is often included as a component in homeopathic complexes used for upper respiratory tract ailments. The efficacy of such remedies for the relief of symptoms or the improvement of quality of life was analysed in clinical studies, which reported positive results^5,9–12^.

Ethanol extracts of *Drosera* species contain high concentrations of flavonoids (hyperoside, isoquercitrin, quercetin, and myricetin-3-O-galactoside) together with phenolic acids (ellagic acid)^13^. Flavonoids have been reported to have anti-inflammatory^14^, antioxidant and antimicrobial properties, which are efficacious in the treatment of respiratory diseases^15^. Traces of naphthoquinones were also found in some preparations^16^.

Bronchial epithelial cells are specialized cells strictly connected to a tissue that form a barrier that protects against external agents of damage. Epithelial membrane integrity could be injured by pathogenic events that damage basal cellular activities (cell cycle, viability, energy balance and oxidative stress) or impair specific activities, such as the maintenance of the electrolytic balance (functioning of membrane ion channels)^17–19^. With appropriate stimulation, the bronchial epithelium can contribute to innate immunity by secreting immune-stimulatory and modulatory mediators, including cytokines, chemokines, growth factors, and lipid mediators that recruit and activate effector cells and antigen-presenting cells^20–22^. Differentiated 16HBE cells simulate many of the activities of the bronchial epithelium and are considered a good model for basic studies.

The aim of this study was to evaluate the effect of a low homeopathic concentration of *D. rotundifolia* ethanolic extract on the transcriptome of the human bronchial epithelial cell line 16HBE. Transcriptomics, which is an important tool of functional genomics research, can be used to study overall gene expression and function, which could be the first step in revealing specific biological processes and molecular mechanisms that are involved in the occurrence of the disease. For this study, a *D. rotundifolia* third decimal (3×) dilution was used (1000 times more diluted than the whole ethanolic extract). The 3× terminology corresponds to the traditional homeopathic nomenclature according to the Anglo-American convention^23^. The goal of the study was to discover the potential gene targets of *D. rotundifolia* 3× after 3 and 6 h of incubation by means of the RNA-seq technique. After validating the main genes using RT-qPCR, the main biological functions involved in *D. rotundifolia* 3× action were identified.

## Results

### Analysis of polyphenols in the *D. rotundifolia* ethanolic extract

The concentration of polyphenols in the *D. rotundifolia* ethanolic extract was estimated to be 7.1 × 10^−3^ mol/L in terms of the gallic acid equivalents. This concentration corresponds to approximately 1.2 mg/mL of total polyphenols, which in turn corresponds to a concentration of 12 µg/mL in the *D. rotundifolia* 3× test sample and 1.2 µg/mL (7.1 × 10^−6^ mol/L) in the final cell culture. The *D. rotundifolia* ethanolic extract was also qualitatively analysed by mass spectrometry. In the HPLC 6.590 min retention time peak, the mass spectrum showed peaks corresponding to ellagic acid (m/z = 300.997905) and isoquercitrin (m/z = 463.087935). In addition, at the 8.004 min retention time peak, quercetin (m/z = 301.035282) was detected. Regarding the presence of hyperoside, which has the same molecular mass as isoquercitrin, there is a chance that the isoquercitrin peak represented a mixture of both compounds. The results confirmed the presence of important bioactive components in *D. rotundifolia* ethanolic extract^13^.

### Effect of *D. rotundifolia* 3× on 16HBE cell viability

**Figure 1:**
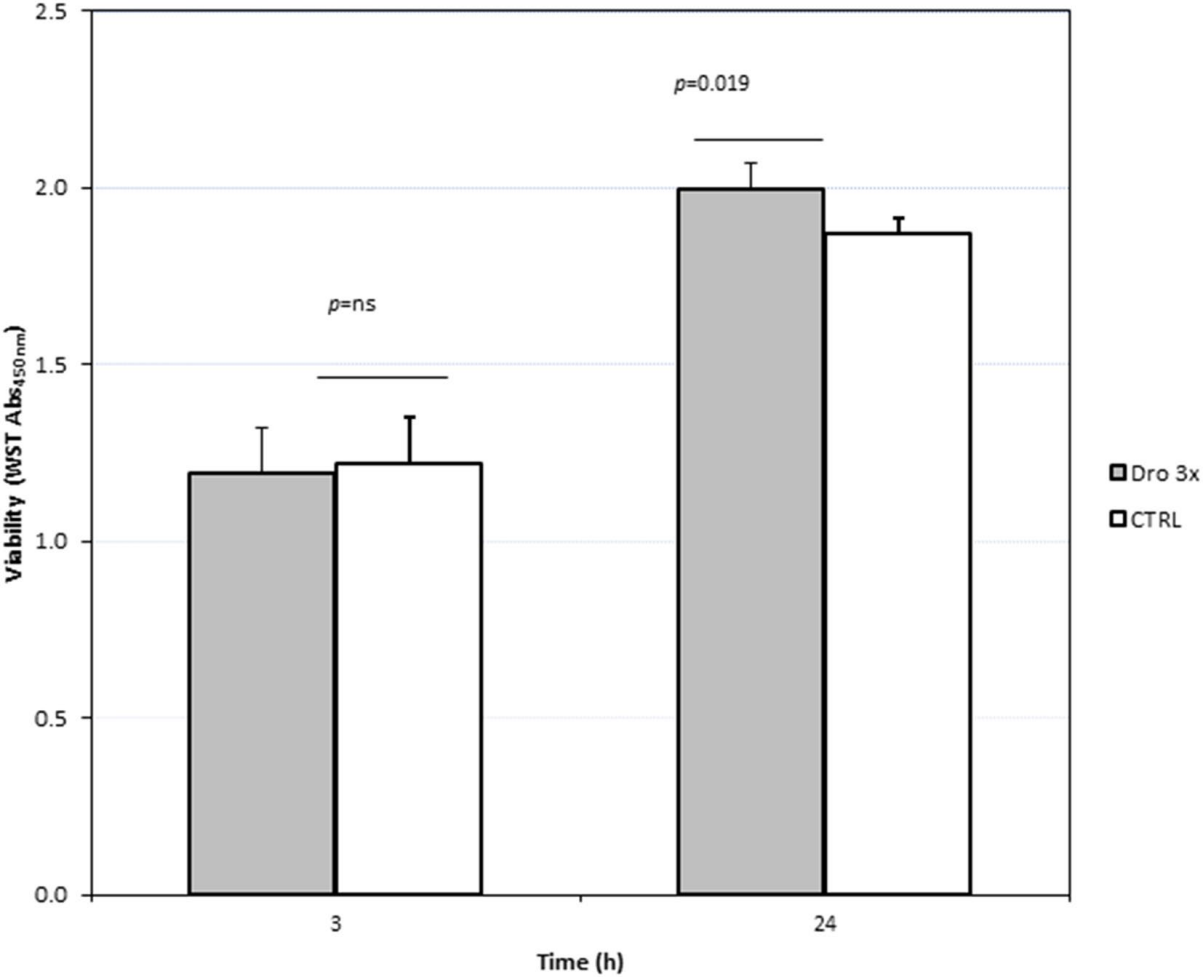
Viability of 16HBE cells incubated with *D. rotundifolia* 3 × or Ctrl for 3 h or 24 h. The colorimetric assay was based on the reduction of the WST reagent as described in the “Materials and method” section.

The viability of the bronchial epithelial cells (16HBE) (Fig. 1) was not impaired after 3 h of exposure to *D. rotundifolia* 3× or the corresponding control (Ctrl), demonstrating the absence of cytotoxicity caused by the plant preparation at the dilutions used in the experiments. Furthermore, after 24 h of incubation with *D. rotundifolia* 3×, the cells showed a small (+ 6.9%) but significant (*p* = 0.019) increase in cell viability. No morphological changes or alteration of cell adherence to the bottom of the well could be observed by optical microscopic inspection (data not shown).

### Changes in gene expression after *D. rotundifolia* 3× treatment

The effects of *D. rotundifolia* 3× treatment on the global gene expression of 16HBE cells were investigated after 3 h of incubation by comparison with the control. The experiments were reproduced in 4 different biological replicates, and the gene expression profile in response to *D. rotundifolia* 3× treatment was investigated by RNA-seq analysis.

The reads obtained by the sequencing were quantified as transcripts by Salmon, which showed an average mapping coverage of 92%. Differential gene expression analysis was then performed to identify the significant target genes of *D. rotundifolia* 3× treatment. The differential expression output obtained from DESeq2 analysis displayed a total of 69 statistically significant (padj < 0.05) differentially expressed genes (DEGs); 44 genes were upregulated (Table 1), and 25 genes were downregulated (Table 2). The lof2FoldChange ranged from + 0.7 (maximum upregulation, Table 1) to − 0.51 (maximum downregulation, Table 2).

**Table 1:** Statistically significant upregulated genes in 16HBE cells treated for 3 h with *D. rotundifolia* 3x.

ENSEMBL ID	Symbol	log2 Fold change	lfcSE	baseMean	padj	Gene description
ENSG00000138061	*CYP1B1*	0.702	0.036	18,306.85	2.5E−79	Cytochrome P450 family 1 subfamily B member 1
ENSG00000163659	*TIPARP*	0.607	0.062	3055.70	7.7E−19	TCDD inducible poly(ADP-ribose) polymerase
ENSG00000124882	*EREG*	0.437	0.069	3248.29	1.5E−07	Epiregulin
ENSG00000115008	*IL1A*	0.436	0.060	3083.65	1.4E−09	Interleukin 1 alpha
ENSG00000197632	*SERPINB2*	0.413	0.062	2963.76	4.0E−08	Serpin family B member 2
ENSG00000260261	*AC124944.3*	0.391	0.063	424.71	2.9E−07	Programmed cell death 6 (PDCD6) pseudogene
ENSG00000118564	*FBXL5*	0.365	0.064	1637.91	1.3E−05	F-box and leucine rich repeat protein 5
ENSG00000109321	*AREG*	0.340	0.059	5103.51	6.4E−06	Amphiregulin
ENSG00000182585	*EPGN*	0.335	0.064	1402.14	1.0E−04	Epithelial mitogen
ENSG00000139289	*PHLDA1*	0.311	0.054	3077.59	7.9E−06	Pleckstrin homology like domain family A member 1
ENSG00000235220	*HLA-F*	0.306	0.062	315.13	4.2E−04	Major histocompatibility complex, class I, F
ENSG00000104361	*NIPAL2*	0.287	0.063	662.15	1.8E−03	NIPA like domain containing 2
ENSG00000119927	*GPAM*	0.277	0.070	1520.61	2.1E−02	Glycerol-3-phosphate acyltransferase, mitochondrial
ENSG00000081041	*CXCL2*	0.273	0.070	525.50	2.8E−02	C-X-C motif chemokine ligand 2
ENSG00000277758	*FO681492.1*	0.266	0.051	96.38	3.2E−05	Synaptotagmin-15
ENSG00000175414	*ARL10*	0.258	0.063	339.66	9.1E−03	ADP ribosylation factor like GTPase 10
ENSG00000120738	*EGR1*	0.256	0.046	3828.16	2.5E−05	Early growth response 1
ENSG00000154639	*CXADR*	0.254	0.062	1339.05	1.4E−02	CXADR Ig-like cell adhesion molecule
ENSG00000087074	*PPP1R15A*	0.252	0.047	3208.72	7.8E−05	Protein phosphatase 1 regulatory subunit 15A
ENSG00000178295	*GEN1*	0.248	0.054	1919.26	2.4E−03	GEN1 Holliday junction 5′ flap endonuclease
ENSG00000139292	*LGR5*	0.247	0.054	102.30	1.8E−03	Leucine rich repeat containing G protein-coupled receptor 5
ENSG00000136244	*IL6*	0.244	0.066	1010.05	5.0E−02	Interleukin 6
ENSG00000005243	*COPZ2*	0.243	0.063	159.54	2.3E−02	COPI coat complex subunit zeta 2
ENSG00000235030	*IER3*	0.233	0.049	3086.81	1.1E−03	Immediate early response 3
ENSG00000111912	*NCOA7*	0.230	0.043	7681.40	8.6E−05	Nuclear receptor coactivator 7
ENSG00000118523	*CTGF*	0.229	0.060	1498.96	3.9E−02	Cellular communication network factor 2
ENSG00000103995	*CEP152*	0.225	0.062	622.41	2.9E−02	Centrosomal protein 152
ENSG00000159086	*PAXBP1*	0.221	0.057	1656.63	2.9E−02	PAX3 and PAX7 binding protein 1
ENSG00000102804	*TSC22D1*	0.212	0.048	2739.98	4.5E−03	TSC22 domain family member 1
ENSG00000108669	*CYTH1*	0.212	0.057	1860.07	5.0E−02	Cytohesin 1
ENSG00000134294	*SLC38A2*	0.208	0.032	11,532.92	1.0E−07	Solute carrier family 38 member 2
ENSG00000103257	*SLC7A5*	0.194	0.030	35,338.50	1.4E−07	Solute carrier family 7 member 5
ENSG00000129474	*AJUBA*	0.181	0.042	5412.41	6.6E−03	Ajuba LIM protein
ENSG00000100644	*HIF1A*	0.180	0.045	6302.38	1.8E−02	Hypoxia inducible factor 1 subunit alpha
ENSG00000138434	*ITPRID2*	0.152	0.039	18,731.03	3.4E−02	ITPR interacting domain containing 2
ENSG00000010310	*GIPR*	0.151	0.036	120.73	2.5E−03	Gastric inhibitory polypeptide receptor
ENSG00000128272	*ATF4*	0.146	0.037	12,347.69	2.4E−02	Activating transcription factor 4
ENSG00000023445	*BIRC3*	0.137	0.037	13,084.30	5.0E−02	Baculoviral IAP repeat containing 3
ENSG00000206489	*PPP1R10*	0.106	0.026	26.98	1.0E−07	Protein phosphatase 1 regulatory subunit 10
ENSG00000278516	*LENG1*	0.050	0.018	12.47	9.7E−04	Leukocyte receptor cluster member 1
ENSG00000061455	*PRDM6*	0.032	0.019	21.63	1.4E−02	PR/SET domain 6
ENSG00000173503	*LTA*	0.028	0.016	15.34	2.9E−02	Lymphotoxin alpha
ENSG00000189068	*VSTM1*	0.027	0.016	24.23	6.6E−03	V-set and transmembrane domain containing 1
ENSG00000275428	*AC024940.6*	0.026	0.016	23.11	3.3E−03	Ovostatin 2

The table reports the gene identification (ENSEMBL ID, gene symbol and description), the degree of the average expression changes for the 4 experiments (log2FoldChange), the standard error of the change (lfcSE), the mean expression value expressed as the normalized read counts (baseMean) and the adjusted p-values (padj) corrected by the Benjamini–Hochberg (BH) method.

**Table 2:** Statistically significant downregulated genes in 16HBE cells treated for 3 h with *D. rotundifolia* 3x.

ENSEMBL ID	Symbol	log2FoldChange	lfcSE	baseMean	padj	Gene description
ENSG00000059804	*SLC2A3*	− 0.510	0.070	2797.19	1.4E−09	Solute carrier family 2 member 3
ENSG00000165507	*DEPP1*	− 0.482	0.070	807.55	2.1E−08	DEPP1 autophagy regulator
ENSG00000116285	*ERRFI1*	− 0.384	0.064	5529.11	2.5E−06	ERBB receptor feedback inhibitor 1
ENSG00000168209	*DDIT4*	− 0.323	0.065	4188.35	3.7E−04	DNA damage inducible transcript 4
ENSG00000146830	*GIGYF1*	− 0.322	0.068	2461.89	1.2E−03	GRB10 interacting GYF protein 1
ENSG00000183691	*NOG*	− 0.303	0.070	430.04	5.2E−03	Noggin
ENSG00000204267	*TAP2*	− 0.297	0.070	824.16	9.1E−03	Transporter 2, ATP binding cassette subfamily B member
ENSG00000213859	*KCTD11*	− 0.284	0.063	1506.09	2.8E−03	Potassium channel tetramerization domain containing 11
ENSG00000169992	*NLGN2*	− 0.273	0.068	1214.66	1.6E−02	Neuroligin 2
ENSG00000171345	*KRT19*	− 0.269	0.050	30,099.26	8.1E−05	Keratin 19
ENSG00000148926	*ADM*	− 0.266	0.070	392.45	3.9E−02	Adrenomedullin
ENSG00000177606	*JUN*	− 0.260	0.062	1999.96	9.1E−03	Jun proto-oncogene, AP-1 transcription factor subunit
ENSG00000145331	*TRMT10A*	− 0.259	0.068	387.61	3.8E−02	tRNA methyltransferase 10A
ENSG00000135636	*DYSF*	− 0.253	0.056	2045.20	2.7E−03	Dysferlin
ENSG00000099308	*MAST3*	− 0.253	0.066	455.28	2.9E−02	Microtubule associated serine/threonine kinase 3
ENSG00000113369	*ARRDC3*	− 0.244	0.059	942.12	1.4E−02	Arrestin domain containing 3
ENSG00000104517	*UBR5*	− 0.235	0.056	3357.96	1.0E−02	Ubiquitin protein ligase E3 component n-recognin 5
ENSG00000186352	*ANKRD37*	− 0.222	0.049	73.34	1.2E−03	Ankyrin repeat domain 37
ENSG00000146674	*IGFBP3*	− 0.204	0.053	22,877.33	3.3E−02	Insulin like growth factor binding protein 3
ENSG00000115524	*SF3B1*	− 0.203	0.039	11,529.03	1.2E−04	Splicing factor 3b subunit 1
ENSG00000070614	*NDST1*	− 0.175	0.036	13,053.04	1.0E−03	N-deacetylase and N-sulfotransferase 1
ENSG00000232070	*TMEM253*	− 0.113	0.028	38.83	1.4E−05	Transmembrane protein 253
ENSG00000241370	*RPP21*	− 0.056	0.019	14.73	5.3E−04	Ribonuclease P/MRP subunit p21
ENSG00000282752	*CTDP1*	− 0.040	0.018	14.79	3.9E−02	CTD phosphatase subunit 1
ENSG00000069188	*SDK2*	− 0.029	0.013	8.71	1.2E−02	Sidekick cell adhesion molecule 2

For the legend see Table 1.

### Gene ontology classification of differentially expressed genes

To better understand the functions of the identified genes, functional analysis was performed by submitting the up- and downregulated gene list to gene ontology (GO) enrichment analysis using the Bioconductor/R package gprofiler2. The representative enriched biological processes and the genes associated with these processes are represented in Table 3. The DEGs were mainly enriched in biological processes, including general functions such as “regulation of response to stimulus”, which includes 34 of the 69 genes, and more specific biological activities, such as “vasculature development” (13 of 69 genes) and “positive regulation of vascular endothelial growth factor production” (5 of 69 genes). A group of 10 genes is associated with the function “epithelial cell proliferation”. This gene set includes the upregulated gene *LGR5* (a GPCR receptor protein and member of the Wnt signalling pathway that controls cell proliferation), the downregulated gene *IGFBP3* (encoding insulin-like growth factor-binding protein 3, which exerts anti-proliferative effects in many cell types) and 8 other genes with specific GO functions related to epithelial regulation (the upregulated epithelial growth factor-like ligands *EREG* and *EPGN*, the downregulated EGF-linked inhibitor *ERRFI1*), proteins involved in cell membrane repair (*DYSF*) or in tissue development (*NOG*) and two transcription factors (*HIF1A* and *JUN*). A group of DEGs is related to the production of cytokines, suggesting their possible role in immunity. Other processes that are significantly associated with the target genes are “regulation of cell death” and “positive regulation of the mitotic cell cycle”, suggesting their involvement in the recovery of epithelial tissue.

**Table 3:** Representative biological processes and associated differentially expressed genes (DEGs) in 16HBE cells treated with *D. rotundifolia* 3 × for 3 h.

GO term	Biological processes	*p*-value	n. genes	DEGs
GO:0010575	Positive regulation of vascular endothelial growth factor production	8.24E−05	5	*CYP1B1, IL1A, HIF1A, ATF4, IL6*
GO:0048583	Regulation of response to stimulus	0.003206	34	*CYP1B1, IL1A, SERPINB2, PPP1R10, EREG, ERRFI1, AREG, EGR1, PPP1R15A, NCOA7, EPGN, DDIT4, HLA-F, IER3, LGR5, GIPR, DYSF, NOG, AJUBA, JUN, UBR5, ARRDC3, CXADR, NLGN2, HIF1A, GPAM, ATF4, LTA, IGFBP3, CTDP1, ADM, BIRC3, CYTH1, IL6*
GO:0050673	Epithelial cell proliferation	0.003256	10	*EREG, ERRFI1, AREG, EPGN, LGR5, DYSF, NOG, JUN, HIF1A, IGFBP3*
GO:0042035	Regulation of cytokine biosynthetic process	0.005926	4	*IL1A, EREG, ERRFI1, EGR1*
GO:1901184	Regulation of ERBB signalling pathway	0.008551	4	*EREG, ERRFI1, AREG, EPGN*
GO:0001944	Vasculature development	0.011299	13	*CYP1B1, TIPARP, IL1A, EREG, ERRFI1, EGR1, EPGN, DYSF, NOG, JUN, HIF1A, ADM, IL6*
GO:0001819	Positive regulation of cytokine production	0.014852	10	*CYP1B1, IL1A, EREG, EGR1, HLA-F, HIF1A, ATF4, LTA, BIRC3, IL6*
GO:0045931	Positive regulation of mitotic cell cycle	0.017961	5	*IL1A, PPP1R10, EREG, EPGN, GEN1*
GO:0050678	Regulation of epithelial cell proliferation	0.02095	8	*EREG, ERRFI1, AREG, EPGN, DYSF, NOG, JUN, HIF1A*
GO:0010941	Regulation of cell death	0.021917	19	*CYP1B1, IL1A, SERPINB2, PPP1R10, PHLDA1, EGR1, NCOA7, DDIT4, HLA-F, IER3, NOG, JUN, HIF1A, ATF4, LTA, IGFBP3, ADM, BIRC3, IL6*
GO:0001568	Blood vessel development	0.038694	12	*CYP1B1, TIPARP, IL1A, EREG, EGR1, EPGN, DYSF, NOG, JUN, HIF1A, ADM, IL6*
GO:0050679	Positive regulation of epithelial cell proliferation	0.049145	6	*AREG, EPGN, DYSF, NOG, JUN, HIF1A*

Table reports enriched biological processes identification (GO term and Biological process name), the number of differentially expressed genes belonging to each biological processes and their gene symbols. *p*-values indicate the enrichment significance obtained with g_SCS algorithm of gprofiler2 analysis, as described in the “Materials and methods” section.

### RT-qPCR validation of the *D. rotundifolia* 3× target genes

For mRNA expression validation, genes that are associated with enriched biological processes and identified by RNA-seq were investigated by RT-qPCR to validate the effects of *D. rotundifolia* 3× on mRNA expression (Table 4). Importantly, genes were selected mainly if they were associated with a biological function (Table 3), and then cut-off values were applied to the genes reported in Tables 1 and 2 as follows: log2FoldChange > 0.1 and <  − 0.1, baseMean > 100 and transcripts per million (TPM) > 1 (data not shown). For many candidate genes, the analysis of their expression based on the experimental series of the RNA-seq study was corroborated by the inclusion of results from 4 new follow-up experiments (for a total of 8 independent samples).

**Table 4:** mRNA expression in 16HBE cells incubated with *D. rotundifolia* 3 × or Ctrl for 3 h.

Gene	n	*D. rotundifolia* 3x (MNE) (mean ± SD)	Ctrl (MNE) (mean ± SD)	Log2FoldChange	SE	*p*-val
***TIPARP***	**4**	**0.0082 ± 0.0008**	**0.0041 ± 0.0003**	**0.982**	**0.149**	**0.013**
***CYP1B1***	**8**	**0.0549 ± 0.0044**	**0.0345 ± s0.0042**	**0.921**	**0.069**	**0.000**
***SERPINB2***	**4**	**0.0004 ± 0.0000**	**0.0002 ± 0.0000**	**0.781**	**0.129**	**0.026**
***EREG***	**8**	**0.0104 ± 0.0025**	**0.0084 ± 0.0031**	**0.574**	**0.099**	**0.037**
***AREG***	**8**	**0.0492 ± 0.0109**	**0.0398 ± 0.0115**	**0.519**	**0.225**	**0.018**
*EPGN*	8	0.0190 ± 0.0041	0.0138 ± 0.0036	0.491	0.157	0.094
*IL1A*	8	0.0121 ± 0.0014	0.0088 ± 0.0006	0.433	0.077	0.337
***CTGF***	**4**	**0.0090 ± 0.0006**	**0.0068 ± 0.0005**	**0.415**	**0.121**	**0.045**
*PPP1R15A*	4	0.0132 ± 0.0015	0.0101 ± 0.0009	0.381	0.162	0.102
*CXCL2*	4	0.0279 ± 0.0041	0.0214 ± 0.0027	0.362	0.16	0.138
*AJUBA*	4	0.0187 ± 0.0019	0.0146 ± 0.0006	0.34	0.142	0.125
***PHLDA1***	**8**	**0.0255 ± 0.0071**	**0.0205 ± 0.0056**	**0.331**	**0.188**	**0.034**
*IL6*	4	0.0110 ± 0.0010	0.0088 ± 0.0003	0.305	0.092	0.067
*EGR1*	8	0.0566 ± 0.0090	0.0472 ± 0.0053	0.239	0.174	0.115
*CXCL8*	4	0.0112 ± 0.0007	0.0097 ± 0.0007	0.222	0.101	0.136
*NCOA7*	4	0.0034 ± 0.0004	0.0030 ± 0.0002	0.177	0.161	0.342
*SASH1*	4	0.0029 ± 0.0003	0.0026 ± 0.0003	0.17	0.204	0.446
*HBEGF*	4	0.0030 ± 0.0004	0.0027 ± 0.0001	0.106	0.095	0.339
*DDIT3*	4	0.0077 ± 0.0004	0.0076 ± 0.0008	0.03	0.128	0.923
*PIK3C2A*	4	0.0030 ± 0.0003	0.0029 ± 0.0002	0.028	0.179	0.801
*NR2F2*	4	0.0482 ± 0.0024	0.0504 ± 0.0067	− 0.035	0.144	0.700
*IGFBP3*	4	0.2175 ± 0.0381	0.2247 ± 0.0377	− 0.039	0.138	0.758
*IRS2*	4	0.0078 ± 0.0003	0.0086 ± 0.0008	− 0.123	0.158	0.474
*JUN*	4	0.0047 ± 0.0006	0.0051 ± 0.0004	− 0.14	0.075	0.200
***ADM***	**4**	**0.0022 ± 0.0002**	**0.0025 ± 0.0001**	− **0.214**	**0.057**	**0.022**
*DDIT4*	8	0.0386 ± 0.0102	0.0514 ± 0.0160	− 0.404	0.248	0.403
*SCL2A3*	4	0.0093 ± 0.0010	0.0123 ± 0.0010	− 0.407	0.176	0.115
***ERRFI1***	**8**	**0.0180 ± 0.0026**	**0.0234 ± 0.0019**	− **0.42**	**0.172**	**0.006**
*DEPP1*	4	0.0058 ± 0.0004	0.0081 ± 0.0007	− 0.478	0.149	0.059
***ARRDC3***	**8**	**0.0040 ± 0.0009**	**0.0067 ± 0.0009**	** − 0.757**	**0.11**	**0.017**

16HBE cells were cultured with *D. rotundifolia* 3 × or the control for 3 h, and their mRNA expression of the indicated genes was evaluated by RT-qPCR. Gene expression is depicted as the mean normalized expression (MNE) after *GAPDH* mRNA normalization (mean ± SD of the indicated number of experiments). *p*-values were calculated by Student’s t test. Values in bold indicate *p*-value < 0.05.

As shown in Table 4, RT-qPCR confirmed the significant changes in the regulation of 10 genes identified by the RNA-seq analysis. The remaining investigated genes showed changes similar to those revealed by RNA-seq (see Tables 1, 2), which were not statistically significant. Notably, the differential expression changes in the relevant genes associated with the enriched biological processes, such as *CY1B1*, *EREG*, *AREG*, *CTGF*, and *ERR1F1*, were verified by RT-qPCR in new follow-up experiments performed with cells treated for 3 h with *D. rotundifolia* 3×.

### Time course of the *D. rotundifolia* 3× effect

**Figure 2:**
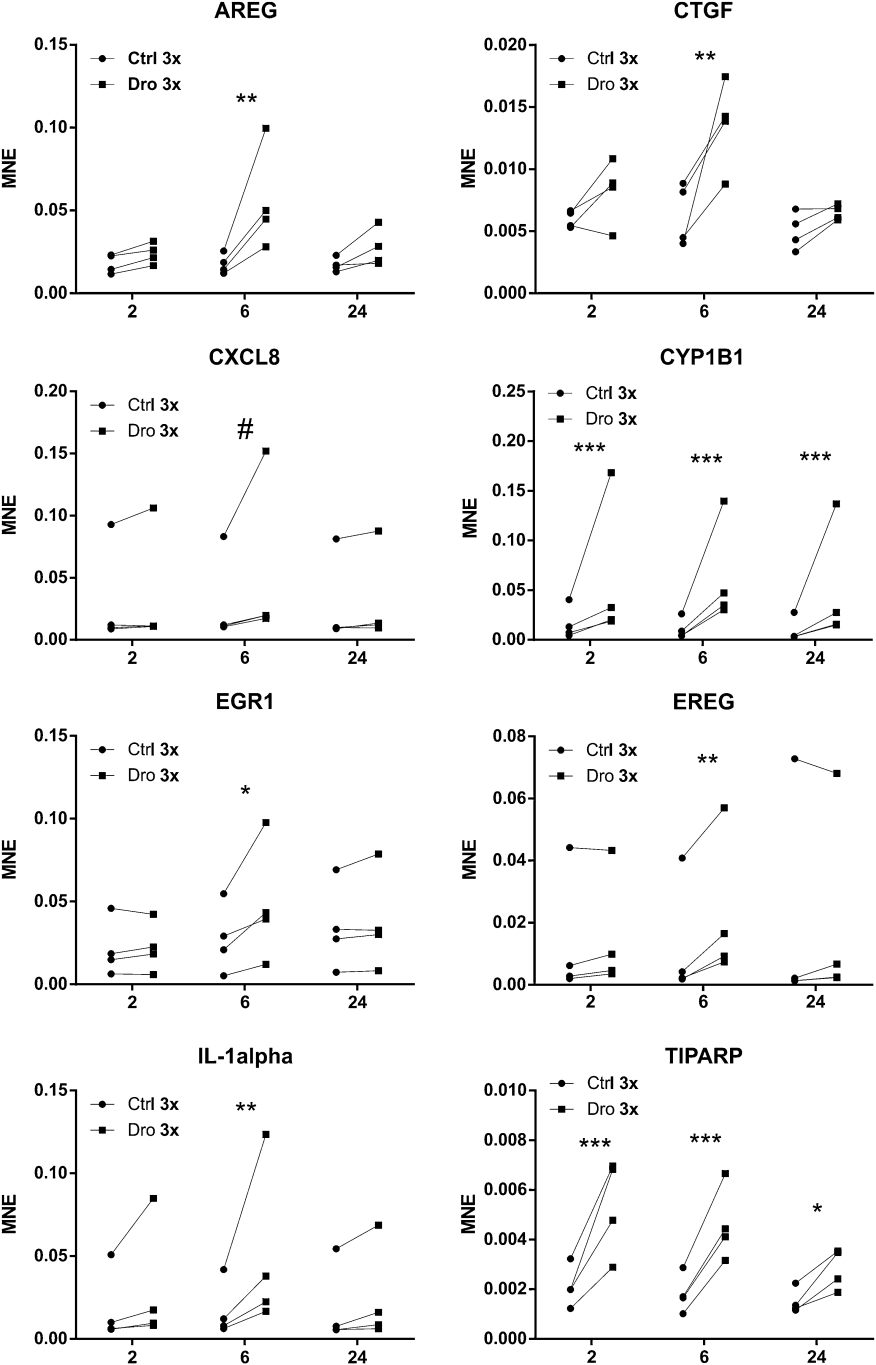
Kinetics of *AREG*, *CTGF*, *CXCL8*, *CYP1B1*, *EGR1*, *EREG*, *IL-1α* and *TIPARP* mRNA expression in 16HBE cells incubated with *D. rotundifolia* 3x. 16HBE cells were cultured with *D. rotundifolia* 3× (Dro 3×) or Ctrl (Ctrl 3×) for up to 24 h to evaluate mRNA expression by RT-qPCR. Gene expression is depicted as the mean normalized expression (MNE) after *GAPDH* mRNA normalization (n = 4). Asterisks indicate a significant increase: *p < 0.05, **p < 0.01, ***p < 0.001, ^#^*p* = 0.057 by two-way ANOVA followed by Bonferroni’s post-test.

Some representative and very well-expressed genes were selected to investigate the kinetics of the effects of *D. rotundifolia* 3× during the time course of the cell treatment. A new series of four experiments was performed, in which the cells were incubated for 2 h, 6 h, and 24 h with the medicine or the control. Figure 2 displays the mRNA expression of *AREG*, *CTGF*, *CXCL8*, *CYP1B1*, *EGR1*, *EREG*, *IL-1α* and *TIPARP* in cells with and without *D. rotundifolia* 3× at the indicated times. The results of all four experiments are separately shown since one of the experiments resulted in higher levels of mRNA expression for a few genes (e.g., *CXCL8*, *EREG* and *IL-1α*). Despite the heterogeneity in the basal gene expression, the direction of the effect is clear-cut, and it is largely significant for at least one time point based on the statistics for the paired data. The experiments showed that the effect of *D. rotundifolia* 3× on most genes starts already at two hours, it is expressed in a higher degree after 6 h, while it decreases at 24 h. Exceptions to this trend are *CYP1B1* and *TIPARP*, which showed the maximum activity at 2 h, which was decreased at 6 h and further decreased at 24 h.

Based on these results, a new RNA-seq analysis of the samples incubated for 6 h with *D. rotundifolia* 3× was performed to describe in detail and under the best conditions all of the effects of the plant on the gene transcription of bronchial cells.

### RNA sequencing of 16HBE cells treated with *D. rotundifolia* 3× for 6 h

RNA-seq was performed using the same parameters used for the RNA-seq of 16HBE cells treated with *D. rotundifolia* 3× for 3 h as well as the bioinformatics analysis. All samples passed the quality control tests as described for the RNA-seq of the 3 h-treated samples.

**Figure 3:**
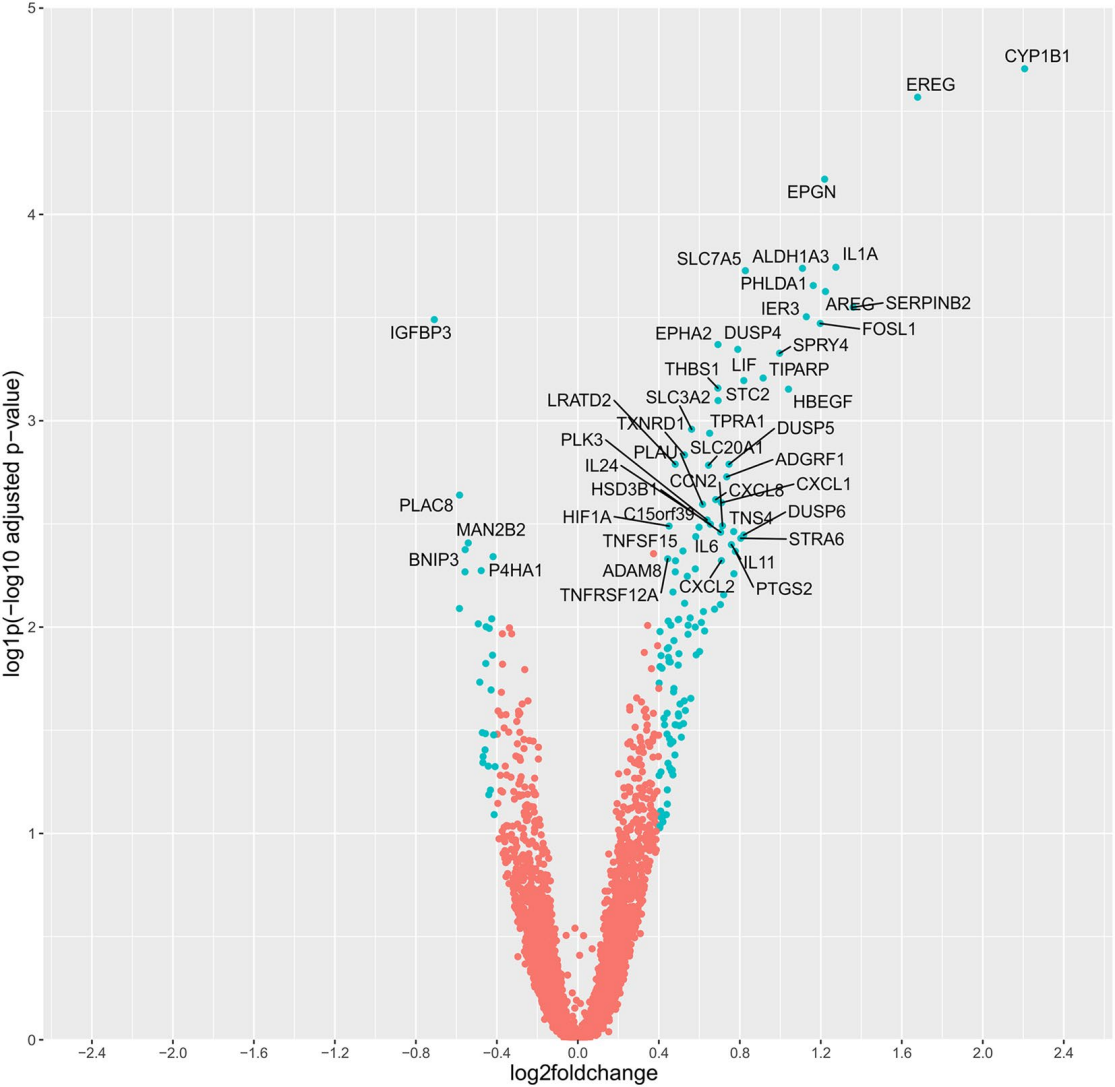
Volcano plot of the differential expression analysis of 16HBE cells treated with *D. rotundifolia* 3× for 6 h. The scattered points represent genes: the x-axis is the log2foldchange for the *D. rotundifolia* 3×-treated 16HBE cells. The y-axis shows the log1p(− log10 adjusted p-value), which better highlights the differentially expressed genes in our experimental conditions. Blue dots represent genes that were significantly differentially expressed (adjusted p-value < 0.05) with a log2foldchange <  − 0.4 or > 0.4 after 6 h treatment with *D. rotundifolia* 3×.

The DESeq2 output generated a list of 495 DEGs in 16HBE cells after 6 h of treatment with *D. rotundifolia* 3× compared to treatment with the control (padj < 0.05), which contains a majority of upregulated genes (n = 334). Figure 3 depicts the DEGs in a Volcano plot, which highlights (blue dots) all the genes (n = 117) that had a statistically significant change (adjusted p-value < 0.05) and a log2FoldChange higher than + 0.4 or less than − 0.4. For this analysis, a log2FoldChange cut-off value was used since the number of DEGs was much higher compared to that identified by the RNA-seq performed for the 3 h treatment. Figure 3 shows that after 6 h of incubation, there were a high number of DEGs, especially upregulated genes, which is in accordance with what is shown in Fig. 2. From a qualitative point of view, a similarity is observed in the genes whose expression is increased at the two different incubation times. Such a similarity is reflected by the upregulation of some genes, such as *CYP1B1*, *EPGN*, *EREG, SERPINB2*, *IL-1α*, *AREG,* and *PHDLA1*, which were also upregulated by cells treated with *D. rotundifolia* 3× for 3 h (see Table 1). Regarding the downregulated genes (left side of Fig. 3), some new genes associated with an inhibitory effect are highlighted, including *IGFBP3*, *PLAC8*, *MAN2B2*, and *BNIP3*, which, except for *IGFBP3*, were identified only after 6 h of incubation and not after a shorter exposure (2 or 3 h). Moreover, genes that were slightly downregulated after 3 h of *D. rotundifolia* 3× treatment, such as *ERRFI1*, were not found to be regulated after 6 h of treatment.

### Functional analysis

**Figure 4:**
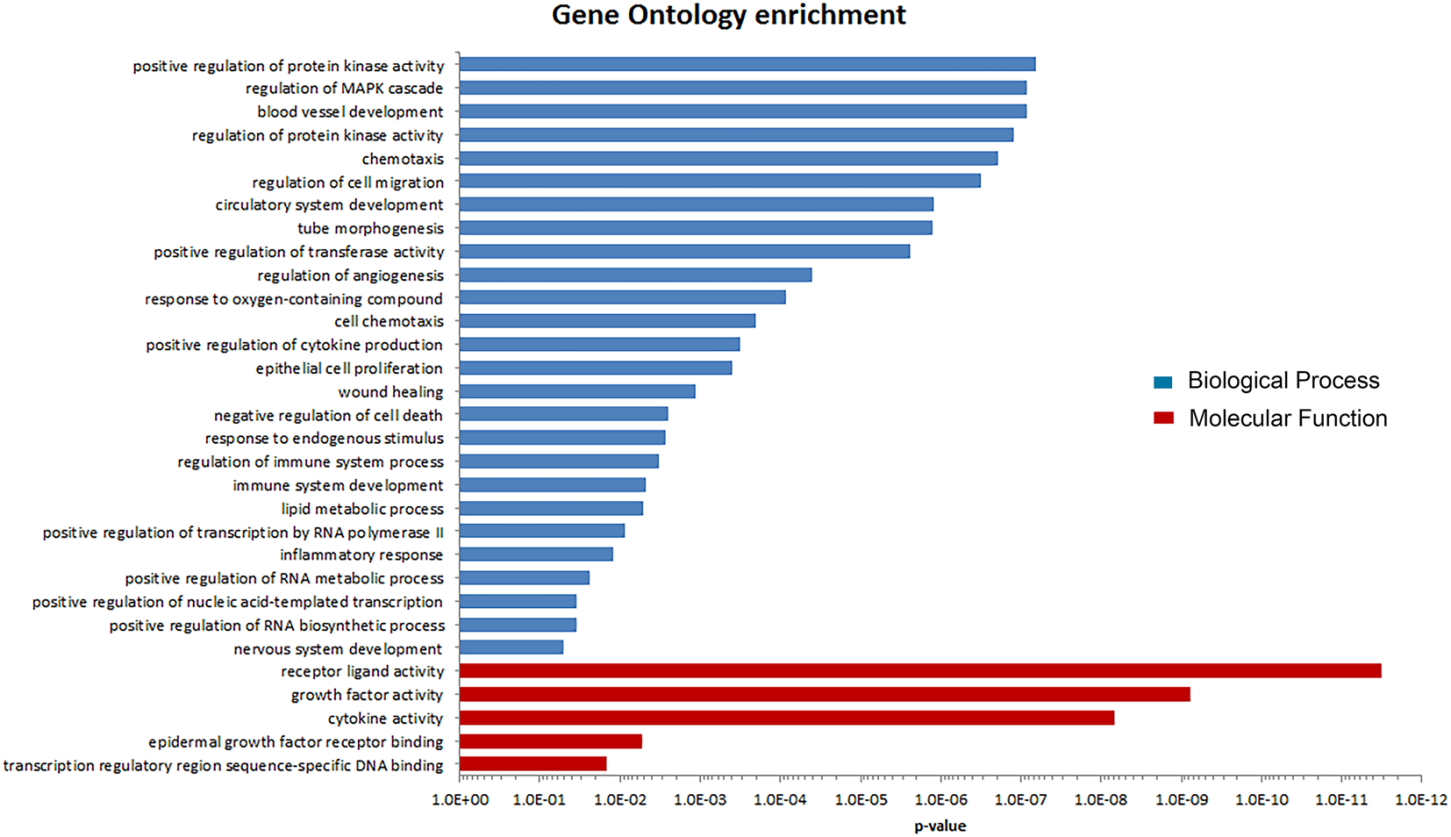
Representative gene ontology classifications of the differentially expressed genes in 16HBE cells treated with *D. rotundifolia* 3×. The differentially expressed genes were classified into two categories: biological processes and molecular functions. Gene ontology enrichment analysis was performed using the “gost” function from the gprofiler2 package according to the gene list ranked by the adjusted *p*-value. GO terms with p-values < 0.05 were considered significant.

The 117 differentially expressed genes were ranked by the adjusted p-value significance, and GO enrichment analysis was performed using gprofiler2 as described in the “Materials and methods” section. The enrichment results for the 6 h data set (Fig. 4) show a wide range of information concerning the biological processes (blue bars in Fig. 4) and molecular functions (red bars). Similar enriched biological processes were observed after 6 h and 3 h of *D. rotundifolia* 3× treatment, such as functions related to blood vessel development, regulation of epithelial cell proliferation and cytokine production. Moreover, there was an increase in biological processes related to inflammation and chemotaxis after 6 h of *D. rotundifolia* 3× treatment due to the upregulation of additional cytokines and chemokines. These data confirm that there is an increase in the effects and suggest new functions associated with *D. rotundifolia* 3× treatment of 16HBE cells after 6 h.

**Figure 5:**
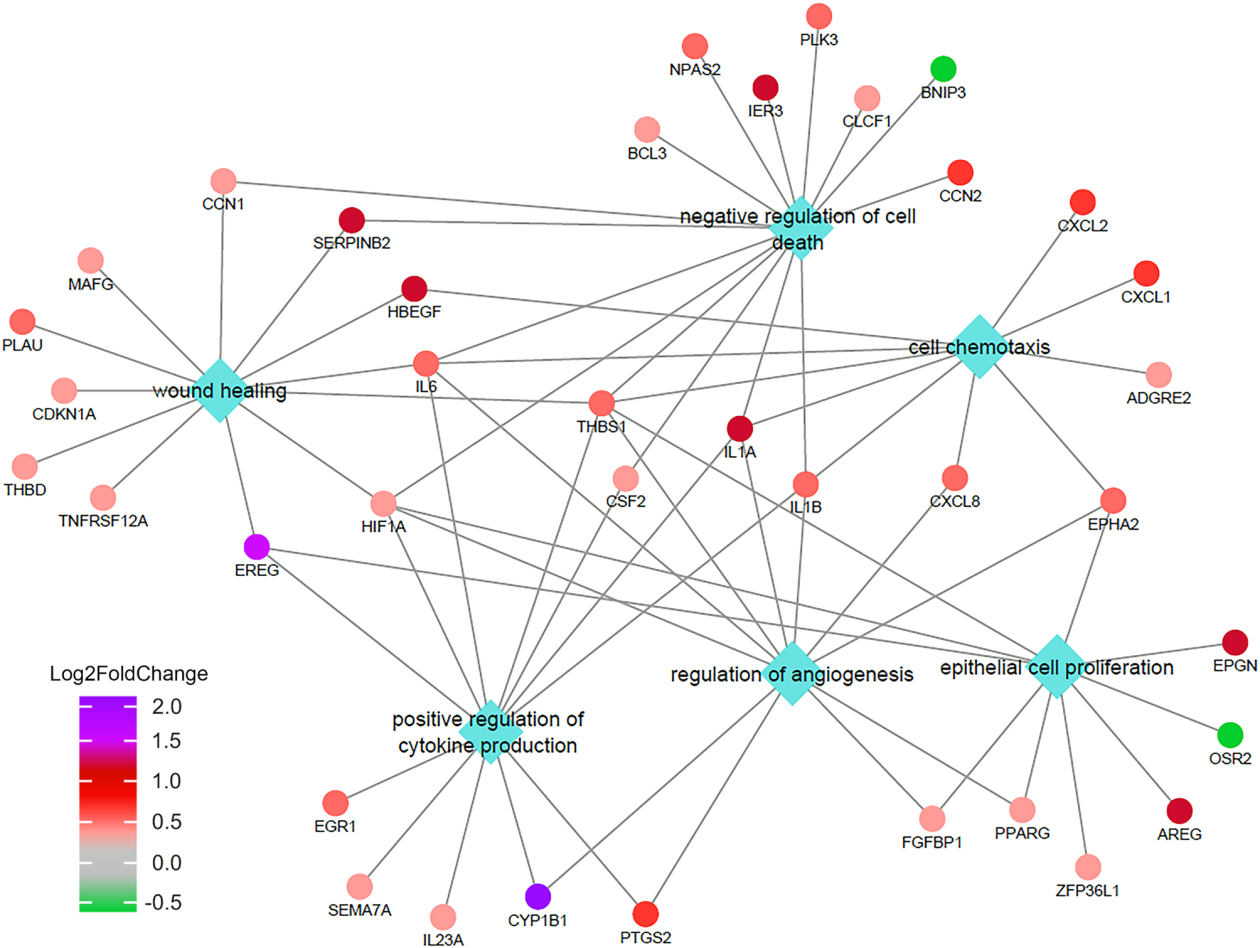
Functional network of enriched biological processes and associated genes. The network was constructed using the selected significant GO terms shown in Fig. 4, which were uploaded into Cytoscape software as described in “Materials and method” section. Diamonds indicate biological processes and circles indicate associated genes.

To draw a functional picture of the enriched functions and their correlations, a network that associates the genes with the functions in the biological model of bronchial epithelial cells was constructed (Fig. 5). Functions are representative and were chosen based on our experimental model and enrichment p-value. The Log2FoldChange values from the DESeq2 output are depicted by the coloured nodes, which shows that the enrichment of these biological functions is mostly associated with the upregulated genes. This was expected since the number of upregulated genes observed after *D. rotundifolia* 3× treatment was almost 3 times higher than the number of downregulated genes. Moreover, genes that were upregulated by *D. rotundifolia* 3× are associated with many biological processes, while this was not the case for the downregulated genes.

## Discussion

In this study, functional genomic research was performed to explore the effects of a medicinal plant traditionally used for respiratory diseases on its potential target cells in vitro, i.e., bronchial epithelial cells. This study shows that a diluted herbal extract used in a traditional homeopathic pharmacopoeia^5,9–12^ can induce a change in mRNA expression that can be measured reliably and reproducibly. Low doses of traditional plants and homeopathic dilutions are now being investigated by means of molecular biology techniques for differential gene expression analysis and bioinformatics^24–28^.

Screening in bronchial cells with RNA-seq analysis showed that low amounts of *D. rotundifolia* at a 3× dilution, which represents a dose commonly used in homeopathic and phytotherapeutic syrups, changed the expression of dozens of genes after 3 h, and this effect was amplified after 6 h of treatment. Validation with RT-qPCR confirmed the differential expression of the genes of interest and showed that after 6 h of *D. rotundifolia* 3× treatment, there was an increase in mRNA expression. Since RT-qPCR sensitivity is higher than that of RNA-seq, such results support the findings of the RNA-seq analysis, suggesting that treatment with *D. rotundifolia* × can be followed-up by the investigation of the expression of these genes.

In our experiments, *D. rotundifolia* 3× did not impair cell viability or adherence, suggesting that this dilution is safe when tested directly in cell culture. The same dilution exerted a stimulatory effect on the expression of several genes, including those of inflammatory cytokines. Furthermore, the transcriptomic analysis and RT-qPCR confirmation suggest that, at the dilution used, *D. rotundifolia* 3× works mainly as a stimulant and not as an inhibitor of cell functions. This result is different from the data reported by others in HMC-1 human mast cells^29^, suggesting the anti-inflammatory effects of the plant. This apparent discrepancy could be due to the difference in the biological model, since mast cells are typical inflammatory cells and were stimulated before treatment with *D. rotundifolia*, while our epithelial cells were treated without previous inflammatory stimuli. However, even more importantly, different doses may be utilized: Fukushima et al*.*^29^ obtained an inhibitory effect by using nondiluted fractions of the whole extract of *D. rotundifolia*, while in the present study, the cells were treated with a 3× dilution (1000 times) of the ethanolic extract.

Paper et al.^30^ reported that extracts of *D. rotundifolia* and other species, tested in hen’s egg model, show efficacy as anti-inflammatory, antispasmodic and antiangiogenic agents. In contrast, the data of our study suggest that treatment with *D. rotundifolia* 3× can trigger a mild inflammatory response in 16HBE cells. The latter effect can be attributed to the increase in the mRNA expression of pro-inflammatory cytokines such as *IL-1α*, *IL-1β* and *IL*-6 and chemokines such as *CXCL1*, *CXCL2* and *CXCL8*. Discrepancies in the data from Paper et al.^30^ could be due to the different models or, again, to the doses applied, since we used a homeopathic dilution of the plant extract, which represents a biological stimulus and does not inhibit key cell functions^24^, as conventional anti-inflammatory agents do. Moreover, it is well known that a mild inflammatory response could be beneficial to the immune system by increasing the apoptosis and clearance of inflammatory cells^31,32^. This concept is fully in agreement with homeopathic theory and tradition, in which low doses of pathogenic substances and/or minimally stressful stimuli trigger endogenous responses of “vital energy”, which eventually lead to healing at the cell, tissue or systemic levels^33–35^. The presence of *CXCL1*, *CXCL2* and *CXCL8*, which are mostly chemoattractants for neutrophils, suggests that treatment with *D. rotundifolia* 3× can help to establish a ready-state immune barrier against pathogens in case of bronchial epithelial tissue damage^36^.

Interestingly, *D. rotundifolia* 3× treatment increased the expression of specific epidermal growth factors, such as *AREG*, *EREG* and *EPGN*, by 16HBE cells. The upregulation of these factors suggests that *D. rotundifolia* 3× plays a positive role in the regulation of cell survival, cell proliferation and wound healing. Moreover, all these factors bind to epidermal growth factor receptor (EGFR) to accomplish their functions. In this context, EGFR is widely expressed on the cells present in the bronchial environment, suggesting that the possible release of these growth factors affects not only the proliferation of bronchial epithelial cells but also that of endothelial cells, fibroblasts and vascular cells. In the case of damage to bronchial epithelial tissue, *D. rotundifolia* 3× could contribute to faster healing of the bronchial microenvironment.

*CYP1B1* was the most highly expressed gene after treatment with *D. rotundifolia* 3× at both time points shown in this study. *CYP1B1* is probably activated as a response to treatment with *D. rotundifolia* 3x, since *CYP1B1* belongs to the cytochrome family and is involved in the metabolism of xenobiotics. Moreover, as shown in Fig. 5, *CYP1B1* is connected to the regulation of angiogenesis and the positive regulation of cytokine production because of its role as an antioxidant and NF-kB regulator^37,38^, respectively.

This study has some limitations since some of the findings are based on bioinformatics analysis, and mRNA expression and functional experiments are required to verify the mechanisms of action and effects of the treatment with *D. rotundifolia* 3×*.* In addition, it is important to observe that after 6 h of *D. rotundifolia* 3× treatment, there was a number of differentially expressed genes with log2FoldChange values ranging from + 0.4 to − 0.4, which were not included in the analysis for this manuscript but could be considered for a future study. Indeed, the effects on gene regulation represent only a first functional step of the action of the plant that we have highlighted, and our hypotheses will have to be confirmed with adequate studies in laboratory animals to investigate the therapeutic potential and the mechanisms of action of this plant.

**Figure 6:**
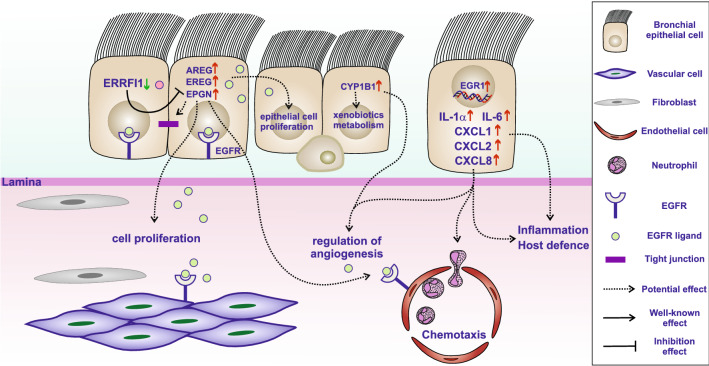
Illustrative graphic summarizing the potential effects of *D. rotundifolia* 3× on bronchial epithelial cells. The presence of *D. rotundifolia* 3× induces the expression of epidermal growth factors (*AREG*, *EREG* and *EPGN*), leading to the growth/repair of bronchial epithelial cells and other cells in the bronchial environment. Those genes, which bind to EGFR to accomplish their functions, are regulated by *ERFFI1*, which is in turn downregulated by *D. rotundifolia* 3× in the first 3 h. *CYP1B1* is highly expressed and is related to the metabolism of xenobiotics and could be involved in the regulation of angiogenesis. Furthermore, inflammatory cytokines (*IL-1α* and *IL-6*) and chemokines (*CXCL1*, *CXCL2* and *CXCL8*) are induced by *D. rotundifolia* 3× and can trigger mild inflammation, increasing chemotaxis and angiogenesis, helping the system in fighting infections.

In conclusion, the data highlight the complex and multifaceted action of the plant on the different layers of the bronchial mucosa, as summarized in Fig. 6. The mRNA expression changes in 16HBE cells treated with *D. rotundifolia* 3× suggest its direct action on the epithelial cell, which protects its integrity with respect to toxic substances (*CYP1B1*) and stimulates its reparative capacity (*AREG*, *EREG* and *EPGN*). In addition, epithelial cells transmit molecular signals that activate mild inflammation (pro-inflammatory cytokines) and recruit innate defence- and angiogenesis-associated cells.

## Materials and methods

### Preparation of *D. rotundifolia* samples

The original round leaf sundew plant, identified with the botanical name *Drosera rotundifolia,* was commercially purchased (Monteagle Herbs, ON, Canada; Document of Authenticity #42808). To the best of our knowledge, no issues related to the Convention on the Trade in Endangered Species of Wild Fauna and Flora have been raised. *Drosera rotundifolia* (ethanolic extract) was prepared by Standard Homeopathic Company, CA, USA. The extract contained 1 g dried whole plant macerated in 10 mL of 45% EtOH, which corresponds to *D. rotundifolia* 1×. Before the experiments, *D. rotundifolia* 1× ethanolic extract was serially diluted 1:10 (0.5 mL + 4.5 mL) twice in sterile pyrogen-free water (B-Braun, Melsungen, Germany) in a 14-mL clean glass tube, which immediately vigorously shaken (succussed) with a Dyna A mechanical shaker that delivered 20 strokes/s for 7.5 s with an 11-mm travel distance. The final solution corresponds to a 3× dilution in 0.45% ethanol solution. The control solution was prepared starting from 45% ethanol (AppliChem, Darmstadt, Germany) fresh solution and succussed as previously described. This was considered the 1× control solution. Two serial decimal dilutions/succussions in ultrapure water were applied as reported above to obtain the control 3× dilution (Ctrl). No filtration was applied at any step. These solutions were used in the culture media at a 1:10 ratio (0.1 mL test solution in 0.9 mL culture medium). Therefore, the final dilution of *D. rotundifolia* was 1000 times greater than that of the ethanolic extract and the ethanol concentration was 0.045%.

### Phenolic quantification

The total phenolic content of the *D. rotundifolia* ethanolic extract was determined by the Folin–Ciocalteu assay^39^. Briefly, 50 μL of extract at an appropriate dilution was mixed with 155 μL of Folin–Ciocalteu reagent diluted 1:10 v/v with water. After 1 min, 40 μL of 20% Na_2_CO_3_ solution was added, and the samples were incubated for 30 min in the dark at 37 °C. The absorbance of each sample was measured at 765 nm. Gallic acid was used as a standard for the calibration curve, and the phenolic content was expressed as gallic acid equivalents. Each determination was repeated three times, and the results are expressed as the mean ± SD.

### Mass spectrometry

A series 1260 HPLC (Agilent Technologies, Waldbronn, Germany) in tandem with a Q-TOF mass spectrometer was used for the present study. The separation was performed on a Zorbax Eclipse XDB (2.1 × 150 mm, 5 µm particle size, Agilent Technologies) with gradient elution. Formic acid (0.1%) was solvent A, and methanol was solvent B; the flow rate was 500 µL/min. An injection volume of 5 µL was used in all experiments. Samples were eluted with a linear gradient from 20 to 95% of solvent B. The MS analysis was carried out using a model 6540 Accurate-Mass Q-TOF LC/MS (Agilent Technologies, Palo Alto, CA, USA) online with HPLC. QTOF-MS was implemented with an electrospray ion source with Agilent Jet Stream technology operating in negative ionization mode. Data acquisition was performed in full scan mode in the mass range of 100–1000 m/z^40^. The standards used were quercetin, ellagic acid (Sigma–Aldrich, Saint Louis, MO, USA), isoquercitrin and hyperoside (HWi, Ruelzheim, Germany).

### Cell treatment

16HBE human bronchial epithelial cells (line 16HBE14o-, kindly provided by Dr Gruenert, University of California, San Francisco) were grown for 1 week in EMEM medium supplemented with 10% FBS (including 2 mM UltraGlutamine, 100 U/mL penicillin and 100 µg/mL streptomycin) from cryogenically frozen samples (2 × 10^6^ cells/vial) prepared from the batch culture (P14, 14th culture passage from the original culture). Fibronectin/collagen/BSA-coated flasks were used^41^. The culture medium was replaced every 3 days. Prior to the treatment, on day 1, cells were seeded in 24-well plates (uncoated) at a density of 0.4 × 10^6^ cells/well in EMEM medium with 2% FBS (including 2 mM UltraGlutamine, 100 U/mL penicillin and 100 µg/mL streptomycin). The plates were incubated until complete adhesion of the cells occurred (16–20 h). On day 2, the culture medium was replaced by 0.9 mL of fresh EMEM with 2% FBS. *D. rotundifolia* 3× or Ctrl was added (0.1 mL/well). The plates were incubated for 2 h, 3 h, 6 h or 24 h depending on each experimental setting. The final volume was 1 mL/well. The Ctrl cell cultures were carried out in parallel with those treated with *D. rotundifolia* for the indicated times in the same culture plates. After the desired incubation period, treated and Ctrl 16HBE cells were collected and subjected to RNA extraction.

### Evaluation of cell viability

Cell viability was checked by the cell proliferation reagent WST-1 (Gibco ThermoFisher Scientific). The WST assay evaluates the cell metabolic activities (NADH reductase) by measuring the chemical modification of the WST tetrazolium salt. 16HBE cells were seeded at a density of 50,000 cells/well in 96-well plates and treated for 3 or 24 h with the *D. rotundifolia* 3× or the Ctrl solutions, which were added to the cell culture at a 1/10 volume ratio. After treatment, 1:10 (v/v) prewarmed WST-1 solution was added to the cells, and the plate was incubated for 90 min. The absorbance (OD) of the samples was measured using a Victor3 multilabel reader (PerkinElmer, Shelton, CT, USA) at 450 nm. Cell viability data were evaluated by t-test statistics (*D. rotundifolia* 3× vs Ctrl).

### RNA extraction

Total RNA from cultured 16HBE cells was isolated using the RNeasy mini Kit (Qiagen, Venlo, Limburg, Netherlands) according to the manufacturer’s instructions. An on-column DNase digestion with the RNase-free DNase kit (Qiagen) was also performed during total RNA isolation to completely remove any possible contaminating DNA. RNA quality and quantity were determined using a Nanodrop 2000 spectrophotometer (Thermo Fisher Scientific, Waltham, Massachusetts, USA)^42^.

### RNA sequencing (RNA-seq)

RNA samples were processed in the Genomic and Transcriptomic Unit at the Technologic Platform Centre (University of Verona, Italy). Total RNA extracted from 16HBE cells was assessed for quality (integrity) using an RNA 6000 Nano Kit (Agilent, Wokingham, UK). The samples with RNA integrity numbers > 9 were considered adequate for library preparation. RNA aliquots (2.5 μg) were used to isolate poly(A) mRNA for the preparation of a directional Illumina RNA-Seq library using the Illumina TruSeq Stranded mRNA Library Prep Kit (Illumina Inc., San Diego, CA, USA). The quality of the library before the generation of the clusters was checked through the visualization of the DNA fragments in the miniaturized electrophoresis system Tape Station (Agilent D1000 Screen Tape system). The average insert sizes were in the range of 300–370 bp. The libraries were also quantified by qPCR using the KAPA Library Quantification kit (Kapa Biosystems Inc., Woburn, MA, USA). The libraries (differentially labelled) were pooled in equal amounts before sequencing. Sequencing was performed with an Illumina NextSeq 500 (Illumina, CA, USA). The samples were sequenced with a single-end protocol (75 base pairs) with a sequence depth of approximately 30 M reads for the sample. After sequencing, the FastQC High Throughput Sequence QC Report (Version 0.11.8) was used to assess the quality of the sequencing based on the FASTQ files.

Transcript quantification was performed with the high-performance computing server at the Computational Unit of the Technologic Platform Centre (University of Verona, Italy). Transcript quantification was determined from the FASTQ reads using the mapping-based mode of Salmon (Version 0.13.1)^43^ with the following parameters: “-i index -l SR -r name -validateMappings -gcBias”. The reference sequence and annotation files were downloaded from the GENCODE repository (Human Release 32, GRCh38.p13) at the following website: https://www.gencodegenes.org/human/. The output of Salmon was then imported into the RStudio environment (R Version 3.5.3; R studio version 1.1.463) using Bioconductor/R package tximport (Version 1.10.10)^44^, which converted data from the transcript level to the gene level during the importing process. Genes with less than 10 reads were discarded from the posterior analysis. Differential expression analysis was performed using the Bioconductor/R package DESeq2 (Version 1.22.2)^45^. After differential analysis, the lfcShrink function in DESeq2 was applied to shrink the log2FoldChanges. Significance values were based on a Wald significance test, and differences in gene expression with an adjusted p-value < 0.05 (corrected by the Benjamini–Hochberg method) were considered significant. For the RNA-seq analysis of the experiments, in which the cells were treated for 6 h with *D. rotundifolia* 3×, a log2FoldChange cut-off value (< 0.4 and > 0.4) was applied. To associate a function with the differentially expressed genes, functional enrichment analysis was performed using the Bioconductor/R package gprofiler2 (Version 0.1.7)^46^. The differentially expressed genes were ranked by the adjusted *p*-value significance, and gene ontology (GO) analysis was queried using the “gost” function in gprofiler2 in “ordered” mode and “g_SCS” as correction/statistical method. The list of significant GO terms was used for the construction of the network using Cytoscape software (Version 3.7.2)^47^.

### Reverse transcription quantitative real-time PCR (RT-qPCR)

Total RNA extracted from 16HBE cells was reverse transcribed into cDNA using the PrimeScript RT reagent Kit (Takara Bio, Kusatsu, Japan), while qPCR was carried out using TB Green Premix Ex Taq (Tli RNase H Plus) (Takara Bio)^42^. The sequences of the gene-specific primers (Thermo Fisher Scientific, Waltham, Massachusetts, USA) used in this study are listed in Table 5. qPCR was performed using a Viia7 Real-time PCR system (Thermo Fisher Scientific). Data were calculated by Q-Gene software (http://www.gene-quantification.de/download.html) and expressed as the mean normalized expression (MNE) units after *GAPDH* normalization^42^. Statistical evaluation was performed by two-way ANOVA followed by Bonferroni’s post hoc test. Values of p < 0.05 were considered statistically significant.

**Table 5:** List of the gene-specific primers used for RT-qPCR.

Gene	Forward	Reverse
*ADM*	AAGTACTTGGCAGATCACTCTC	CCCACTTATTCCACTTCTTTCG
*AJUBA*	CTTTCTACAGTGTCAATGGCTC	CATTGCTTGTAGGATCTTCTCCA
*AREG*	GAGCCGACTATGACTACTCAG	CTTAACTACCTGTTCAACTCTGAC
*ARRDC3*	GTTTATCACTTCCTGAAAGACCTG	CTCTCAAAGTCATCACAAGCAC
*CTGF*	CCCAGACCCAACTATGATTAGAG	CTCCACAGAATTTAGCTCGGT
*CXCL2*	TTTATTTATTTGTTTGTTTTAGAAG	CTAACTTGGGTTTGACCTAAAAT
*CXCL8*	AGAGACAGCAGAGCACACAAG	ACACAGTGAGATGGTTCCTTC
*CYP1B1*	CTGGATTTGGAGAACGTACCG	TCAGGATACCTGGTGAAGAGGA
*DDIT3*	AGTCATTGCCTTTCTCCTTCG	TGATTCTTCCTCTTCATTTCCAGG
*DDIT4*	GAGGAAGACACGGCTTACCT	CAGTAGTTCTTTGCCCACCT
*DEPP1*	CTCATCCATTCTCCTGCCAC	GTGCCAGTCGAGATATAGACC
*EGR1*	AGCAGCAGCACCTTCAACC	GCAGGCTCCAGGGAAAAG
*EPGN*	ATTCAACGCAATGACAGCAC	GCTATGGGTCCTTCTATGTTGTC
*EREG*	ACAGCTTTAGTTCAGACAGAAGAC	GCAAACAATAGCCATTCATGTCAG
*ERRFI1*	CTAATACCACTTGGGCATGCT	AACTTGATCCTCTTCATGTGGTC
*GAPDH*	AACAGCCTCAAGATCATCAGC	GGATGATGTTCTGGAGAGCC
*HBEGF*	CTCATGTTTAGGTACCATAGGAG	CAGTCTGAAATCACCTTGTGTC
*IGFBP3*	ACACTGAATCACCTGAAGTTCC	AGCTCCACATTAACCTTGCG
*IL1A*	AGAGAGGGAGTCATTTCATTGG	ACTCAGAGACACAGATTGATCC
*IL6*	GGCACTGGCAGAAAACAACC	GCAAGTCTCCTCATTGAATCC
*IRS2*	CATCGTGAAAGAGTGAAGATCTG	AAACAGCACAATGATGAATGCC
*JUN*	ACCTTATGGCTACAGTAACCC	TTGCTGGACTGGATTATCAGG
*NCOA7*	GAAGAAGATGGTGGTTCAGAAG	TCAGTGCTATGGAGTTTAGGG
*NR2F2*	GTTCACCTCAGATGCCTGTG	CAGTAACATATCCCGGATGAGG
*PHLDA1*	ATCCACATCCACACTCTCATC	CTTCCTCAAGTCCTCAAAACC
*PIK3C2A*	AGACTCTTGCCATTACAGAATCAG	CTCCAAACAAAGAAGTCACATCAG
*PPP1R15A*	GGGAAGTCAATTTGCAGATGG	CGGTGTGATGGTGGATAAGAG
*SASH1*	TGAAGACGAGGAGAAACCCA	GGTCGCTGTTACTGTCATACTC
*SCL2A3*	GGATGAGCTTTGTCTGTATTGG	CTAAATAGTGAGCAGCGGAGG
*SERPINB2*	GAGGAGAGGAGATTGAAACAATGG	GGGAGAGGAAGAGGTTCTGG
*TIPARP*	CCACAATTCATTCTTCAGGAGAG	CCACCAAGTGTCTGTAAATATGGA

### Ethics approval and consent to participate

Not applicable, as the study did not involve patients, volunteers or animals.

## Data Availability

The raw and processed RNA-seq data are available in the public GEO database (https://www.ncbi.nlm.nih.gov/geo/) under the accession number GSE144215.
